# CED-10/Rac1 Regulates Endocytic Recycling through the RAB-5 GAP TBC-2

**DOI:** 10.1371/journal.pgen.1002785

**Published:** 2012-07-12

**Authors:** Lin Sun, Ou Liu, Jigar Desai, Farhad Karbassi, Marc-André Sylvain, Anbing Shi, Zheng Zhou, Christian E. Rocheleau, Barth D. Grant

**Affiliations:** 1 Department of Molecular Biology and Biochemistry, Rutgers University, Piscataway, New Jersey, United States of America; 2 Division of Endocrinology and Metabolism, Department of Medicine, McGill University and McGill University Health Centre Research Institute, Montreal, Quebec, Canada; 3 Department of Biochemistry and Molecular Biology, Baylor College of Medicine, Houston, Texas, United States of America; National Heart, Lung, and Blood Institute, United States of America

## Abstract

Rac1 is a founding member of the Rho-GTPase family and a key regulator of membrane remodeling. In the context of apoptotic cell corpse engulfment, CED-10/Rac1 acts with its bipartite guanine nucleotide exchange factor, CED-5/Dock180-CED-12/ELMO, in an evolutionarily conserved pathway to promote phagocytosis. Here we show that in the context of the *Caenorhabditis elegans* intestinal epithelium CED-10/Rac1, CED-5/Dock180, and CED-12/ELMO promote basolateral recycling. Furthermore, we show that CED-10 binds to the RAB-5 GTPase activating protein TBC-2, that CED-10 contributes to recruitment of TBC-2 to endosomes, and that recycling cargo is trapped in recycling endosomes in *ced-12*, *ced-10*, and *tbc-2* mutants. Expression of GTPase defective RAB-5(Q78L) also traps recycling cargo. Our results indicate that down-regulation of early endosome regulator RAB-5/Rab5 by a CED-5, CED-12, CED-10, TBC-2 cascade is an important step in the transport of cargo through the basolateral recycling endosome for delivery to the plasma membrane.

## Author Summary

When cargo is internalized from the cell surface by endocytosis, it enters a series of intracellular organelles called endosomes. Endosomes sort cargo, such that some cargos are sent to the lysosome for degradation, while others are recycled to the plasma membrane. Small GTPase proteins (Rabs) are well-known master regulators of endosome function. As cargo moves through the endosomal system, it must pass from the domain controlled by one Rab-GTPase to the domain controlled by another. Little is known about how transitions along the recycling pathway are controlled, or if Rab transitions are necessary for cargo recycling. Here we identified a group of proteins that act on recycling endosomes to deactivate the early acting GTPase RAB-5. Disruption of any of these proteins interferes with recycling. Our work shows that RAB-5 deactivation is important for cargo recycling, and it provides some of the first mechanistic insight into how changes in Rabs can be controlled during endocytic recycling. Importantly, several proteins that we found contribute to this recycling function have roles in other cellular processes, such as cell migration and the removal of cell corpses. Therefore our work also suggests that endocytic recycling could contribute to these processes in previously unsuspected ways.

## Introduction

The *C. elegans* intestine has proven to be a powerful model system for the study of epithelial cell membrane trafficking mechanisms. The worm intestine is a simple epithelial tube consisting of 20 enterocyte cells that form nine “donut-like” intestinal rings [1]. Each of these 20 cells is terminally differentiated, and each intestinal cell is maintained for the life of the animal without replacement [1].

Like mammalian intestinal epithelial cells, *C. elegans* enterocytes display apicobasal polarity with defined apical junctions separating the apical and basolateral domains [1]. The apical enterocyte membranes, which form the intestinal lumen, display a prominent microvillar brush border, with an overlying glycocalyx and underlying subapical terminal web rich in actin and intermediate filaments [1]. The basolateral membrane is in contact with the pseudocoelom (body cavity) and is responsible for the exchange of molecules between the intestine and other tissues of the body.

In previous studies we established three model transmembrane cargo markers for the analysis of basolateral endocytic trafficking in the *C. elegans* intestine: hTAC-GFP, hTfR-GFP, and MIG-14-GFP [2]–[6]. hTAC (human IL-2 receptor alpha-chain) enters cells via clathrin-independent endocytosis (CIE), while hTfR (human transferrin receptor) and MIG-14 (Wntless) enter cells via clathrin-dependent endocytosis (CDE) [7]–[9]. However, while hTAC and hTfR recycle via the recycling endosome in an RME-1/EHD-dependent manner, MIG-14 recycles via retrograde recycling to the Golgi in a retromer-dependent manner [2], [5], [8]–[12]. Thus comparison of the effects of any endocytic transport mutant on these three cargo proteins can give insight into which steps in receptor traffic are affected.

Here we focus on the function of the *C. elegans* Rac1 homolog CED-10 in regulation of epithelial cell endocytic trafficking. During engulfment of dead apoptotic cells, CED-10 functions in a pathway with associated proteins CED-12/ELMO, CED-5/DOCK180, and CED-2/CrkII, promoting cytoskeletal reorganization that is thought to be important for pseudopod formation/function [13]. CED-12 and CED-5 form a bipartite guanine-nucleotide exchange factor for CED-10, and are thus thought to promote conversion of inactive CED-10(GDP) to active CED-10(GTP) [14]. CED-2 physically associates with CED-5 and is thought to function as an adapter, potentially linking the protein complex to certain apoptotic corpse receptors such as MOM-5/Frizzled and/or Integrins, but not the CED-1 corpse receptor [15], [16].

Mammalian Rac1 has been reported to become GTP-loaded on endosomes, and to require Arf6-dependent recycling for membrane ruffling and its localization to the leading edge of migrating cells [17]–[20]. Despite the known association of activated Rac1 with early and recycling endosomes, little is known of the potential role of Rac1 in regulating endosome function. In this study we define an important requirement for CED-10/Rac1 in basolateral recycling in the intestinal epithelia. This function of CED-10/Rac requires CED-5 and CED-12, but not CED-2. Furthermore we connect this recycling function of CED-10 to Rab-GAP TBC-2, indicating a mechanism for the down-regulation of the RAB-5 GTPase as endocytic cargo reaches the recycling endosome.

## Results

### Loss of CED-10/Rac1, CED-12/Elmo, or CED-5/Dock180 leads to intracellular accumulation of recycling cargo

In order to determine if CED-10/Rac1 is required for endocytic transport, we assayed the effect of a strong Rac1 loss-of-function mutant, *ced-10(n3246)*, on the subcellular distribution of three endocytic cargos, using confocal microscopy in the adult intestine [15]. The *C. elegans* intestine is known to express CED-10/Rac1 at high levels, but CED-10/Rac1 function has not been previously investigated in this tissue [21].

**Figure 1:**
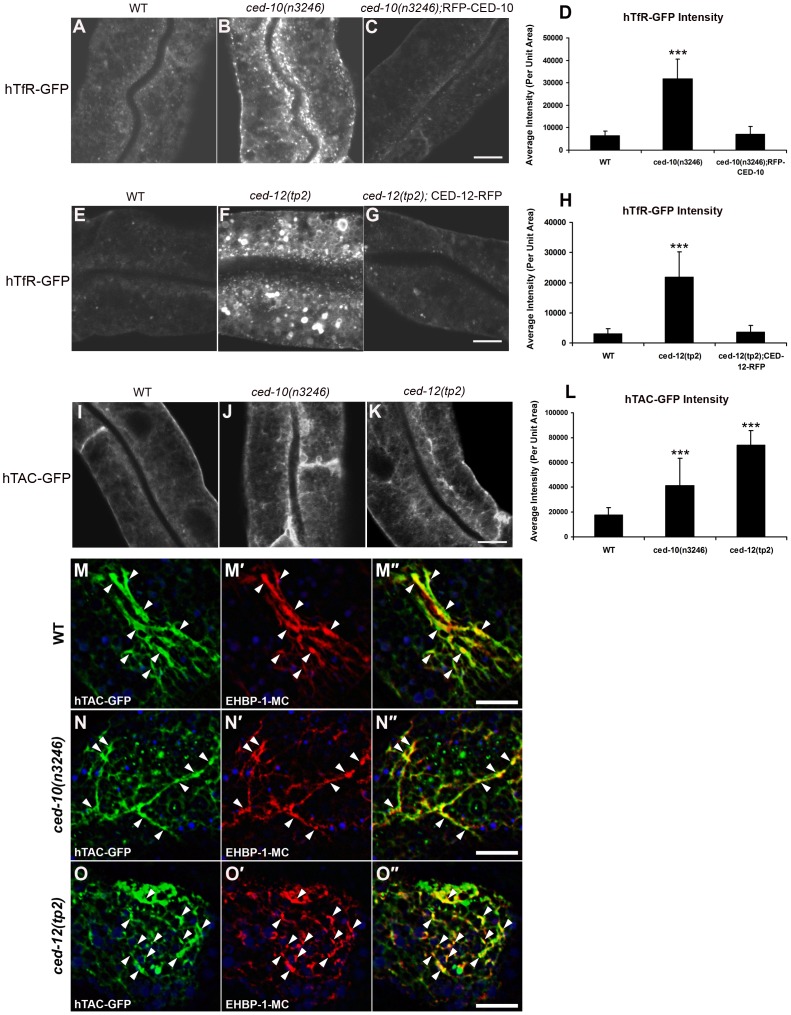
*ced-10 and ced-12* mutants display abnormal trafficking of recycling cargo in the *C. elegans* intestine. (A–C) Confocal images of the worm intestine in live intact animals expressing a GFP-tagged CDE cargo protein that recycles via the recycling endosome, the human transferrin receptor (hTfR-GFP). The *ced-10* mutant phenotype is rescued by intestine-specific expression of RFP-CED-10. (D) Quantification of hTfR-GFP puncta intensity. (E–G) Intracellular hTfR-GFP accumulates in *ced-12(tp2)* mutants. The CED-12 mutant phenotype is rescued by intestine-specific expression of CED-12-RFP. (H) Quantification of hTfR-GFP puncta intensity. (I–K) Representative confocal images of the worm intestine in living intact young adult animals expressing a GFP-tagged CIE cargo protein that recycles via the recycling endosome, the IL-2 receptor alpha chain (hTAC-GFP). Wild type (N2), *ced-10(n3246)*, and *ced-12(tp2)* mutant animals are shown. (L) Quantification of hTAC-GFP intensity. (M–O″) Confocal images of the worm intestine in live intact animals expressing GFP-tagged IL-2 receptor alpha chain (hTAC-GFP) and mCherry-tagged *C. elegans* EHBP-1 (EHBP-1-MC), a recycling endosome marker. Wild-type animals (M–M″), *ced-10(n3246)* mutant animals (N–N″), and *ced-12(tp2)* mutant animals (O–O″) are shown. Error bars represent standard deviations from the mean (n = 18 each, 6 animals of each genotype sampled in three different regions of each intestine). Asterisks indicate a significant difference in the one-tailed Student's T-test (***p<0.0001). Scale bar, 10 µm.

Interestingly, we observed strong intracellular accumulation of recycling receptor hTfR-GFP in the *ced-10* mutant background, similar to that we had previously observed in known endocytic recycling mutants such as *rme-1* and *amph-1* (Figure 1A and 1B, quantified in 1D) [2], [4]. The abnormal intracellular accumulation of hTfR-GFP in the *ced-10(n3246)* mutant was completely rescued by transgenic expression of CED-10 using an intestine-specific promoter, indicating that the effect of CED-10 on recycling is cell autonomous and is not mediated by indirect effects via other tissues (Figure 1C and 1D). We also observed abnormal accumulation of another recycling cargo protein, hTAC-GFP, in *ced-10* mutants, and found that accumulated intracellular hTAC-GFP colocalized with recycling endosome marker EHBP-1 (Figure 1I, 1J, 1L, and 1N–1N″) [6]. TAC and TfR are thought to be internalized independently, meet in the endosomal system, and then recycle from the recycling endosome to plasma membrane in separate carriers [3], [22]–[24]. Taken together our results indicated trapping of multiple types of cargo in the recycling arm of the endocytic pathway in *ced-10*/Rac1 mutants.

Furthermore, we found that *ced-12(tp2)* and *ced-5(n1812)* mutants displayed the same aberrant intracellular accumulation of hTfR-GFP and hTAC-GFP found in *ced-10* mutants, indicating that the CED-12/CED-5 Rac exchange factor complex is also required for the recycling process (Figure 1E, 1F, 1H, 1K and 1L; Figure S2A–S2D and S2I). The abnormal intracellular accumulation of hTfR-GFP in the *ced-12* mutant was completely rescued by transgenic expression of CED-12 using an intestine-specific promoter, again indicating an intrinsic requirement for CED-12 in the intestinal cells (Figure 1G and 1H).

Importantly, *ced-10* and *ced-12* mutants had no effect on the subcellular localization of MIG-14-GFP, indicating that CED-10/Rac1 and CED-12/ELMO are somewhat cargo specific in their effects, and are not required for retrograde recycling from endosomes to the Golgi (Figure S1A–S1G). Since MIG-14 shares its uptake route with hTfR, but is not thought to enter the recycling endosome, these results suggest that CED-10/Rac1 is required for specific membrane trafficking events associated with recycling endosome dependent cargo.

No defect in hTAC-GFP or hTfR-GFP localization was found in *ced-2(e1752)* mutants, indicating that CED-2 is not required for the endocytic transport of these cargos (Figure S3A–S3D, S3I). Since CED-2 is required for phagocytic dead cell engulfment, this result indicates that the observed defects in endocytic traffic are not indirect effects of failed phagocytosis. This is also consistent with previous studies of the *C. elegans* intestinal cells, indicating that after embryogenesis the intestinal cells are not involved in the clearance of apoptotic cell corpses, do not perform phagocytosis, and do not migrate [1], [25]. Our results showing that CED-2 is not required in the intestine for hTfR and hTAC trafficking also indicates that not all CED-10 associated factors are shared between phagocytic and endocytic regulation.

### Loss of CED-10, CED-12, or CED-5 disrupts endosome morphology

**Figure 2:**
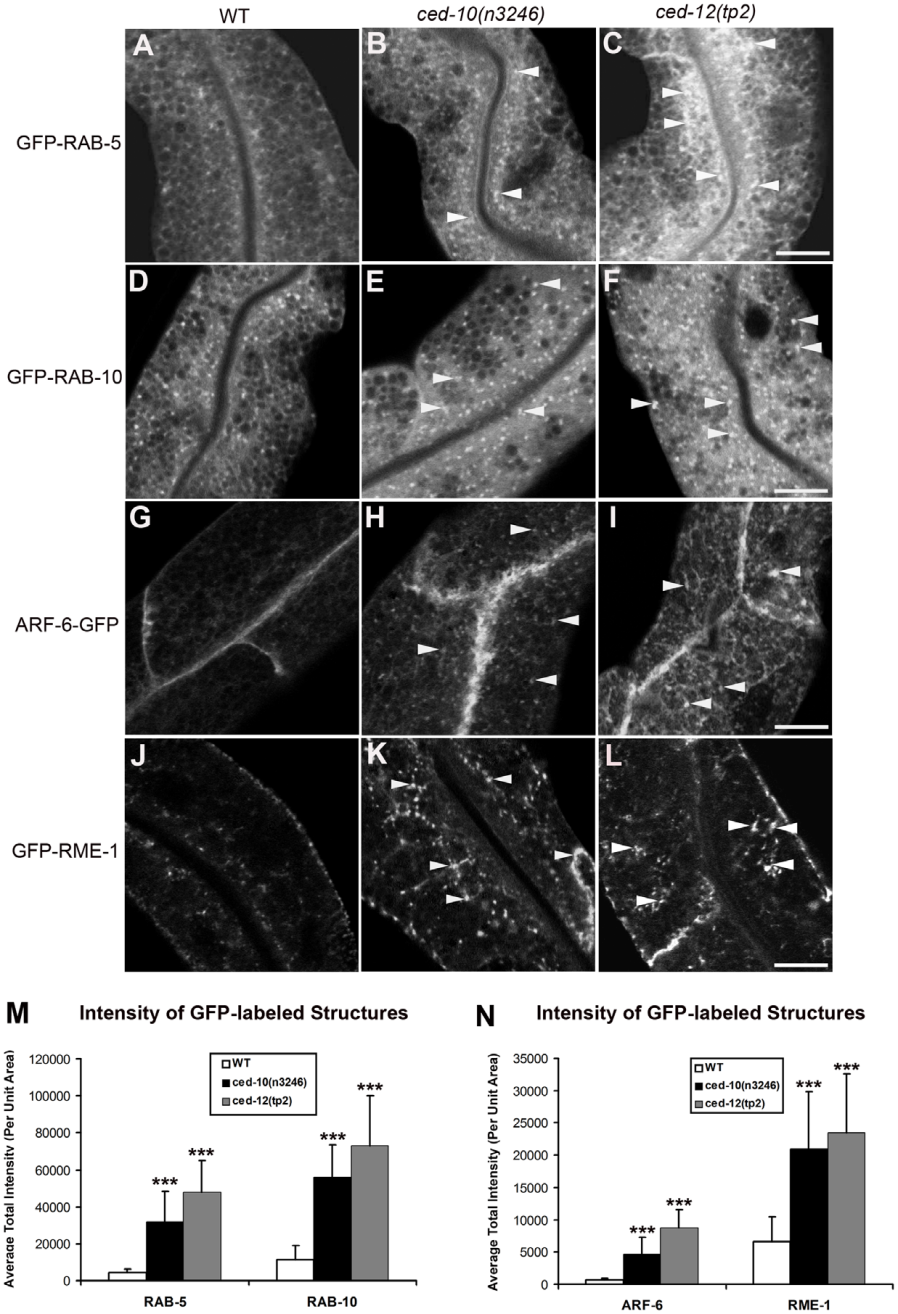
Abnormal accumulation of early and recycling endosomes markers in *ced-10(n3246)* and *ced-12(tp2)* mutants. Representative confocal images are shown for GFP-RAB-5 (A–C), GFP-RAB-10 (D–F), ARF-6-GFP (G–I), and GFP-RME-1 (J–L). Quantifications of average puncta intensity are shown in (M–N). All images were collected from living intact young adult animals expressing GFP-tagged proteins specifically in the intestinal epithelial cells. Error bars represent standard deviation from the mean (n = 18 each, 6 animals of each genotype sampled in three different regions of each intestine). Asterisks indicate a significant difference in the one-tailed Student's t-test (***p<0.0001). Scale bar represents 10 µm.

In order to help determine which step in trafficking is affected by loss of CED-10/Rac1 and its exchange factor CED-5/CED-12, we performed morphometric analysis of a wide-variety of marker proteins associated with endocytic organelles in the intestine of *ced-10* and *ced-12* mutant animals. This set of markers has been used successfully in previous studies to gain insight into the specific defects associated with endocytic transport mutants [2]–[6]. We noted over-accumulation of GFP-tagged early endosome regulator RAB-5 in *ced-10* and *ced-12* mutants (Figure 2A–2C, quantified in 2M and Figure S4). The average GFP-RAB-5 puncta intensity increased by about 8-fold and 12-fold, respectively, in *ced-10* and *ced-12* mutants (Figure 2M). We also noted in both mutants abnormal morphology of basolateral recycling endosomes labeled by GFP-RAB-10, ARF-6-GFP, GFP-RME-1, and SDPN-1-GFP (Figure 2D–2L, quantified in 2M, 2N, Figure S4, and Figure S5A–S5C, quantified in S5J). By contrast, markers for late endosomes (GFP-RAB-7), and apical recycling endosomes (GFP-RAB-11) were unperturbed (Figure S5D–S5I and S5K). We found that *ced-5* mutants, but not *ced-2* mutants, also displayed defective recycling endosome morphology (Figures S2E–S2H, S3E–S3H, S2J and S3J). Taken together with the cargo accumulation results, these specific changes in endosomal morphology indicate that a particular branch of the endocytic pathway, including the early endosomes and basolateral recycling endosomes, but not late endosomes or apical recycling endosomes, require CED-10/Rac1 activity for their normal function.

### CED-10 and CED-12 are associated with early and recycling endosomes

**Figure 3:**
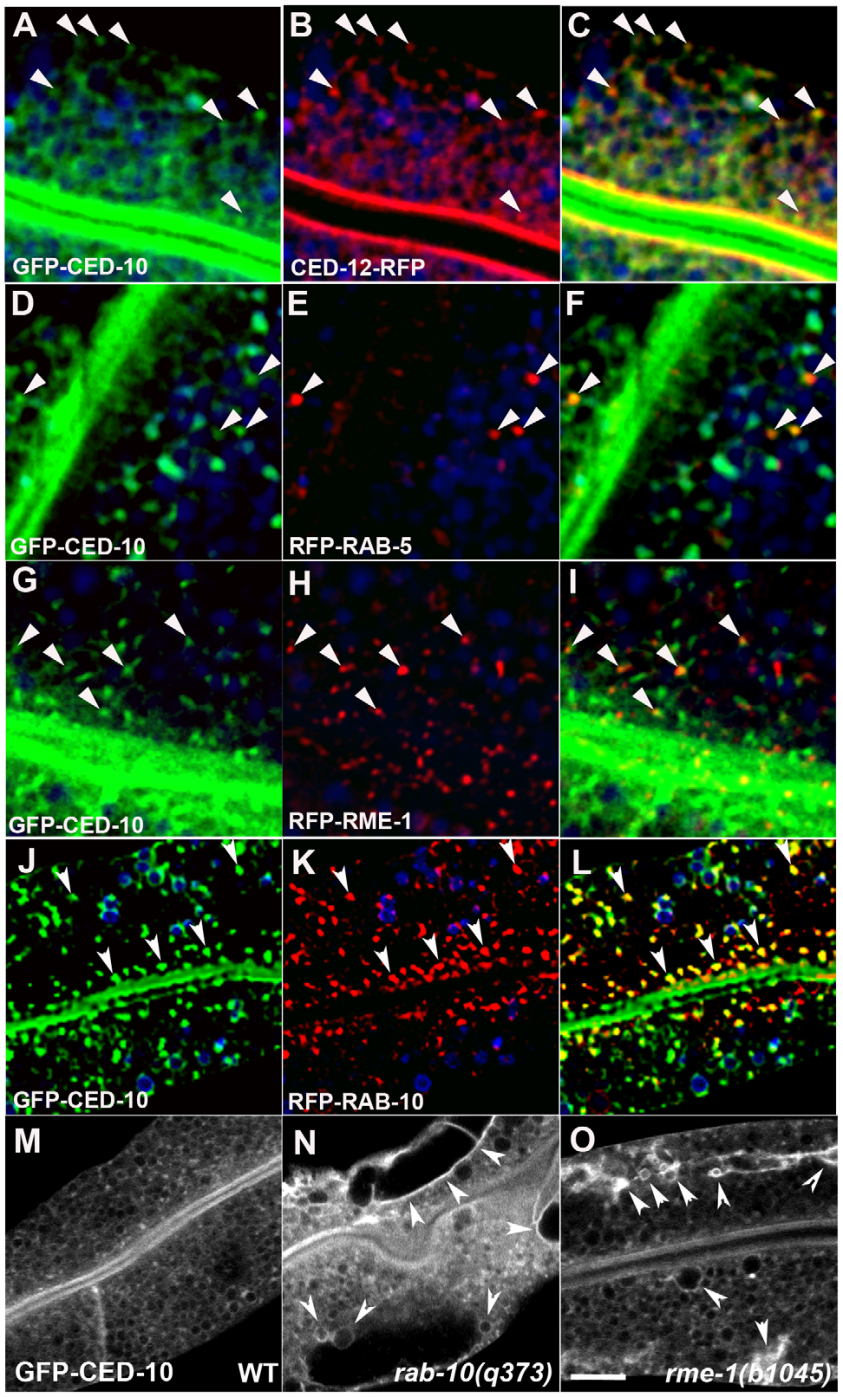
CED-10 localizes to early and recycling endosomes, and colocalizes with CED-12, in the intestine. All images are from wide-field deconvolved, or confocal, 3-D image stacks acquired in intact living animals expressing GFP and RFP tagged proteins specifically in intestinal epithelial cells. (A–C) GFP-CED-10 colocalizes with CED-12-RFP on intracellular puncta. Arrowheads indicate structures labeled by both GFP-CED-10 and CED-12-RFP. (D–F) GFP-CED-10 colocalizes with RFP-RAB-5 on a subset of early endosomes. Arrowheads indicate endosomes labeled by both GFP-CED-10 and RFP-RAB-5. (G–I) GFP-CED-10 partially colocalizes with RFP-RME-1 on basolateral recycling endosomes. Arrowheads indicate endosomes labeled by both GFP-CED-10 and RFP-RME-1. (J–L) GFP-CED-10 colocalizes extensively with RFP-RAB-10 on recycling endosomes. Arrowheads indicate endosomes labeled by both GFP-CED-10 and RFP-RAB-10. (M–O) GFP-CED-10 localizes to the abnormally enlarged endosomes present in *rab-10* and *rme-1* mutants. Arrows mark the grossly enlarged early and recycling endosomes. In each image autofluorescent lysosome-like organelles are shown in blue in all three channels, whereas GFP appears only in the green channel and RFP only in the red channel. Signals observed in the green or red channels that do not overlap with signals in the blue channel are considered bone fide GFP or RFP signals, respectively. Scale bar, 10 µm.

If CED-10 and CED-12 function directly in endosomal regulation, then we would expect to find them associated with endosomes in wild-type cells. Thus we sought to determine the subcellular localization of CED-10 and CED-12 in the intestinal epithelial cells using functional tagged forms of the proteins. GFP-CED-10 localized strongly to the apical domain (likely to the microvilli) and to intracellular puncta in the cytoplasm (Figure 3A). CED-12-RFP colocalized well with GFP-CED-10 to the intracellular puncta (Figure 3B and 3C). However, CED-12-RFP also strongly labeled a subapical band that displayed significant overlap with the GFP-CED-10 labeled apical band (Figure 3A, 3B and 3C). It is not clear if the partial apical overlap represents the presence of both proteins on certain subapical structures, or rather represents two distinct apical localizations for CED-10 and CED-12 that are very near one-another. The simplest interpretation of these results is that the functionally important site of CED-10/CED-12 interaction for the recycling of basolateral cargo is on the intracellular puncta (endosomes – see below), although we cannot exclude important interactions on other subcellular compartments.

We identified the intracellular puncta labeled by CED-10 as endosomes by performing a series of colocalization studies with a previously established set of intestine-specific compartment markers [2]–[6]. CED-10 appeared specifically enriched on endosomes along the early and recycling pathway. We observed direct overlap of intestinal GFP-CED-10-labeled puncta and a subset of early endosomes marked by RFP-RAB-5 (Figure 3D, 3E and 3F). GFP-CED-10 showed the strongest colocalization with recycling endosome marker RFP-RAB-10 (Figure 3J, 3K and 3L), and displayed less overlap with later acting recycling endosome protein RFP-RME-1 (Figure 3G–3I). Similarly, we also observed colocalization between CED-12-GFP and markers of early endosomes and recycling endosomes (Figure S6J–S6L, and data not shown). Little overlap was observed between GFP-CED-10 and markers for late endosomes (GFP-RAB-7), the Golgi (MANS-GFP), or multi-vesicular bodies (GFP-HGRS-1/Hrs) indicating specificity in endosome-type associated with CED-10 (Figure S6A–S6I).

In order to confirm the endosomal localization of CED-10 we assayed for changes in the localization of GFP-CED-10 in *rab-10* and *rme-1* mutant backgrounds where the morphology of specific types of endosomes is specifically disrupted. Indeed we found that loss of either RAB-10 or RME-1 disrupted GFP-CED-10 localization (Figure 3M–3O). In *rab-10* and *rme-1* mutants, GFP-CED-10 labeled the grossly enlarged endosomes that were produced (Figure 3M–3O). Since our previous work showed that *rme-1* mutants accumulate enlarged basolateral recycling endosomes without affecting early endosomes, and *rab-10* mutants accumulate enlarged early endosomes and display reduced numbers of recycling endosomes, our results further indicate the residence of CED-10/Rac1 on both early and recycling endosome types [2], [3].

### TBC-2 functions with CED-10 to mediate recycling

While most Rac1 effectors are thought to be plasma membrane localized, recent work identified the TBC-domain Rab-GAP protein Armus (TBC1D2) as an endosome associated Rac1 effector [26]. This work showed that active GTP-bound Rac1 binds to Armus, regulating the trafficking of E-cadherin, and thus cell adhesion, in MDCK cells [26]. The *C. elegans* homolog of Armus is TBC-2, a TBC-domain protein recently shown to function as a RAB-5 GAP important for the regulation of endocytosis and phagocytosis *in vivo* [27], [28]. Genetic analysis indicated that in the absence of TBC-2, RAB-5 activity is abnormally high, and early and late endosomes of the intestine are enlarged [27]. The enlargement of late endosomes in *tbc-2* mutants could be phenocopied by expression of constitutively active RAB-5(Q78L), and could be suppressed by depletion of RAB-5, RAB-7, or the HOPS complex. Thus it was proposed that hyperactive RAB-5 in *tbc-2* mutants leads to increased RAB-7 activity, and apparent increased lysosomal degradative activity [27], [29].

Previous studies did not determine if TBC-2/Armus is important for endocytic recycling. Since Rab GTPases generally function sequentially as cargo progresses along membrane trafficking pathways, one might expect that de-activation of the early acting GTPase RAB-5 is required to allow proper functioning of recycling endosomes associated with later acting GTPases such as RAB-10 [30]. Thus we sought to determine if TBC-2 functions with CED-10/Rac1 in *C. elegans*, and if TBC-2 function is required for endocytic recycling.

**Figure 4:**
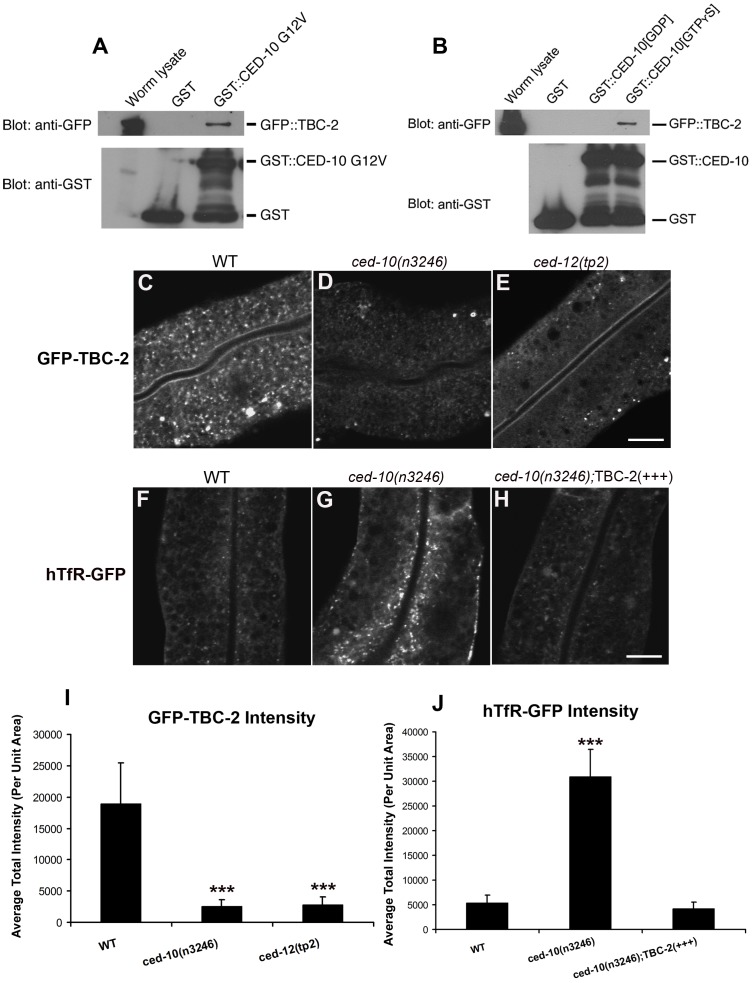
TBC-2 is recruited to endosomes by CED-10. (A) Glutathione beads loaded with recombinant GST or GST-CED-10(G12V), or (B) GST-CED-10(GDP) or GST-CED-10(GTPγS) were incubated with worm lysates containing GFP::TBC-2 and then washed to remove unbound proteins. Bound proteins were eluted and analyzed by Western blot using anti-GFP (top) or anti-GST (bottom) antibodies. Worm lysate represents 1% of the input. No GFP::TBC-2 was detected in non-transgenic worm lysates (not shown). (C–G) Representative confocal micrographs for GFP-TBC-2 and hTfR-GFP in various genetic backgrounds are shown. All images were collected from living intact adult animals expressing intestine-specific transgenes. (C–E) GFP-TBC-2 localization to endosomes is strongly reduced in *ced-10* and *ced-12* mutants. (F–H) hTfR-GFP overaccumulation in *ced-10* mutants is suppressed by over-expression of RFP-TBC-2. (I–J) Quantification of average puncta and tubule intensities for the indicated genotypes are shown. Asterisks indicate a significant difference in the one-tailed Student's T-test (***p<0.0001). For all the presented data, error bars represent standard deviations from the mean (n = 18 each, 6 animals of each genotype sampled in three different regions of each intestine). Scale bar, 10 µm.

In agreement with the work on mammalian Armus, we observed clear and consistent interactions between GFP::TBC-2 and CED-10 in GST pull-down experiments from *C. elegans* lysates. We found that GFP::TBC-2 interacts with a constitutively active mutant form of CED-10, G12V, or wild-type CED-10 loaded with GTPγS, but not wild-type CED-10 loaded with GDP (Figure 4A and 4B). Thus TBC-2 interacts specifically with activated CED-10, indicating that the physical interaction between Rac1/CED-10 and Armus/TBC-2 is evolutionarily conserved.

To determine if CED-10 is important for TBC-2 recruitment to endosomes, we examined the subcellular localization of GFP-TBC-2 in *ced-10* and *ced-12* mutants. We found that the normal punctate endosomal distribution of GFP-TBC-2 was disrupted in animals lacking CED-10 or CED-12 (Figure 4C–4E). The intensity of GFP-TBC-2 endosomal labeling was reduced by 6 to 7-fold in *ced-10* and *ced-12* mutants (Figure 4I). These results indicate that CED-10 and CED-12 are required *in vivo* for the efficient recruitment of TBC-2 to endosomal membranes, likely through direct binding of CED-10(GTP) to TBC-2.

**Figure 5:**
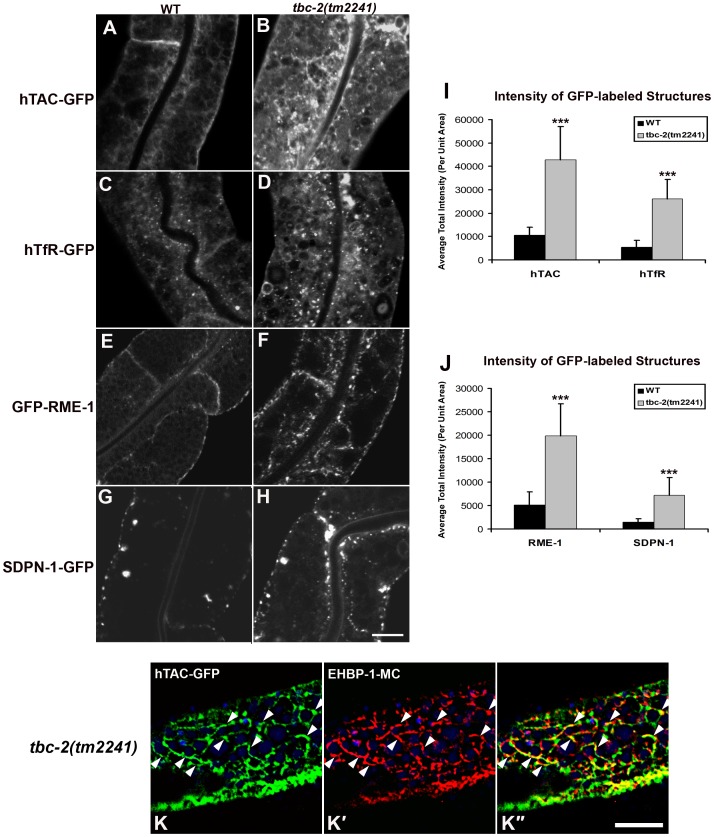
TBC-2 is required for recycling endosome morphology and function. Representative confocal micrographs are shown for hTAC-GFP, hTfR-GFP, GFP-RME-1 and SDPN-1-GFP, in a *tbc-2* mutant background. All images were collected from living intact adult animals expressing intestine-specific transgenes. (A–D) Recycling cargos hTAC-GFP and hTfR-GFP over-accumulate in *tbc-2* mutants. (I) Quantification of hTAC-GFP and hTfR-GFP puncta and tubule intensity in the intestine of living intact wild-type and *tbc-2* mutant animals. (E–H) Recycling endosome markers GFP-RME-1 and SDPN-1-GFP also over-accumulate in *tbc-2* mutants. (J) Quantification of puncta and tubule intensity of GFP-RME-1 and SDPN-1-GFP per unit area. (K–K″) Confocal images of the intestinal epithelium in live intact *tbc-2(tm2241)* mutant animals expressing recycling cargo hTAC-GFP and recycling endosome marker EHBP-1-MC. Autofluorescent lysosomes are shown in blue. Asterisks indicate a significant difference in the one-tailed Student's *t* test (***p<0.0001). Error bars represent standard deviations from the mean (n = 18 each, 6 animals of each genotype sampled in three different regions of each intestine). Scale bar, 10 µm.

**Figure 6:**
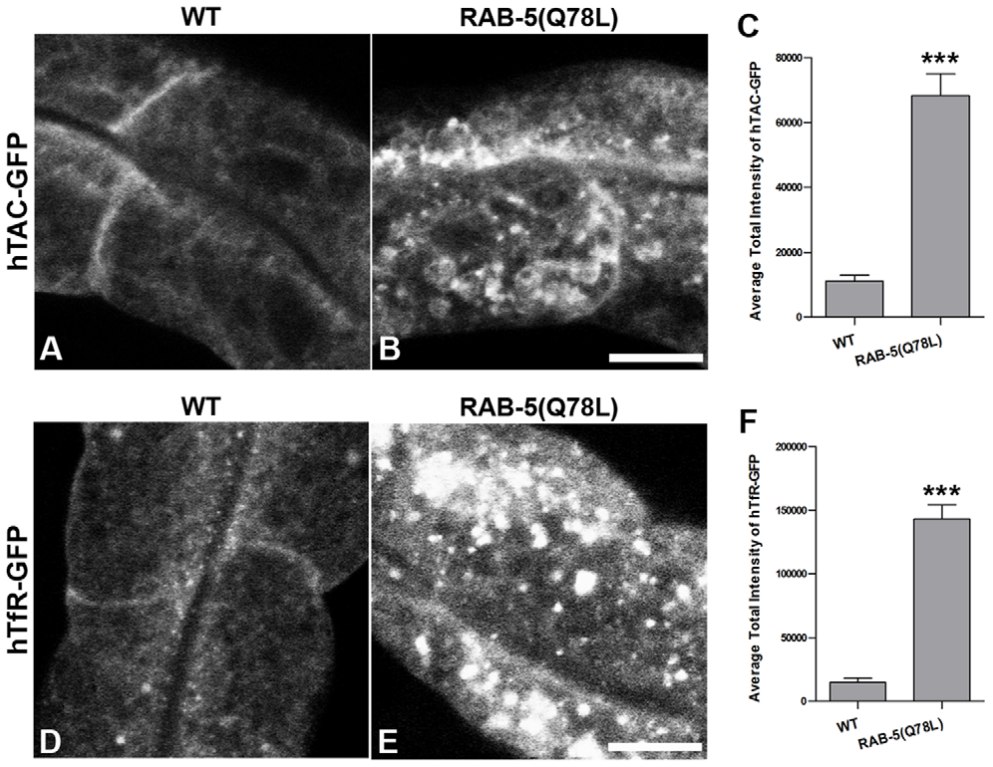
Expression of GTPase-defective RAB-5 interferes with the trafficking of recycling cargo. (A and B) Confocal images of recycling cargo hTAC-GFP in the intestinal epithelium. Wild-type animals (A) and animals expressing of GTPase-defective RAB-5 (tagRFP-RAB-5(Q78L)) (B) are shown. (C) Quantification of hTAC-GFP puncta and tubule intensity. (D and E) Confocal images of recycling cargo hTfR-GFP in the intestinal epithelium. Wild-type animals (D) and animals expressing of GTPase-defective RAB-5 (tagRFP-RAB-5(Q78L)) (E) are shown. (F) Quantification of hTfR-GFP puncta intensity. Error bars represent standard deviations from the mean (n = 18 each, 6 animals of each genotype sampled in three different regions of each intestine). Asterisks indicate a significant difference in the one-tailed Student's T-test (***p<0.0001). Scale bar, 10 µm.

To determine if TBC-2 is important for endocytic recycling, we analyzed the localization of recycling endosome markers and recycling cargo markers in the *tbc-2(tm2241)* deletion mutant. We found that basolateral recycling endosome markers GFP-RME-1 and SDPN-1-GFP were severely perturbed in *tbc-2* mutants in a manner similar to that found in *ced-10*, *ced-12*, and *ced-5* mutants (Figure 5E–5H and 5J). Loss of TBC-2 also resulted in intracellular accumulation of hTAC-GFP and hTfR-GFP, similar to the phenotype observed in *ced-10*, *ced-12*, and *ced-5* mutants (Figure 5A–5D and 5I). hTAC-GFP in *tbc-2* mutants colocalized with recycling endosome marker EHBP-1-MC, indicating cargo trapping in recycling endosomes (Figure 5K–5K″). Furthermore, we reasoned that if the role of TBC-2 in recycling was to convert RAB-5(GTP) to RAB-5(GDP), then expression of GTPase-defective RAB-5 should also interfere with cargo recycling. Consistent with this model we found strong intracellular accumulation of hTAC-GFP and hTfR-GFP in the intestinal cells of animals expressing GTPase-defective RAB-5(Q78L) (Figure 6A–6F). Taken together these results indicate that the recycling of clathrin-independent cargo hTAC and clathrin-dependent cargo hTfR require TBC-2-depedent RAB-5 down-regulation. These results indicate that TBC-2 function is critical for the basolateral endocytic recycling pathway.

We also sought further evidence that TBC-2 functions downstream of CED-10/Rac1 in the recycling pathway. We reasoned that if the *ced-10* recycling phenotype is due to poor recruitment of TBC-2 to endosomes, then overexpression of TBC-2 might ameliorate *ced-10* mutant defects. Indeed, we found that the overexpression of RFP-tagged TBC-2 completely rescued the abnormal accumulation of recycling cargo hTfR-GFP in *ced-10* mutant animals (Figure 4F–4H and 4J). These results strongly suggest that TBC-2 is a key CED-10/Rac1 effector required for endocytic recycling.

## Discussion

Rab conversion has recently emerged as a general principle of membrane traffic, acting as a key regulator of vectorial transport of cargo along the secretory and endocytic pathways [30]. Countercurrent cascades of Rab GEFs and Rab GAPs have been proposed to mediate such Rab conversion [30]. In the simplest form of such a cascade, the early acting Rab recruits the GEF for the next Rab along the pathway, while the later acting Rab recruits the GAP for the earlier acting Rab, producing a directional exchange of Rab-GTPases as cargo progresses along the pathway.

One well-studied Rab conversion event occurs during the early to late endosome transition, and during phagosome maturation, via a RAB-5 to RAB-7 switch [31]–[33]. RAB-5(GTP) recruits the SAND-1(Mon1)/CZZ-1 heterodimer, which in turn acts to displace RABX-5 (Rabex-5), a key RAB-5 GEF [32], [33]. SAND-1/Mon1 also aids in RAB-7 recruitment, in part through interactions with the HOPS complex, a shared RAB-5/RAB-7 effector [32]. Recent evidence in yeast further indicates that the Mon1/Czz1 dimer is a Ypt7 (Rab7) GEF, acting to activate Ypt7 (Rab7) as the endosome matures [34].

TBC-2 also appears to play a role in this process as a RAB-5 GAP. *tbc-2* null mutants produce grossly enlarged early and late endosomes, a phenotype very similar to that produced upon expression of constitutively active RAB-5(Q78L) [27]. These *tbc-2(-)* phenotypes can be suppressed by partial knockdown of RAB-5, RAB-7, or components of the HOPS complex, suggesting that TBC-2 is normally required to dampen the RAB-5 driven cascade that activates RAB-7 [27].

Importantly, our current study shows that TBC-2 also strongly influences the recycling arm of the endocytic pathway, through interaction with CED-10/Rac1. This is particularly interesting because little is known of how the transition from early endosome to recycling endosome is achieved or how Rab GEFs and GAPs might be involved. Most studies on the early endosome to recycling endosome transition have focused on the joint Rab5/Rab4 effector Rabaptin5, or the neuron-specific Rab4 effector GRASP-1 [35], [36]. Our results indicate that CED-10/Rac1 resides on early and recycling endosomes where it is likely activated by the CED-5/CED-12 bipartite GEF. This is reminiscent of the activation of Rac1 by a different Rho-GEF, Tiam1, on endosomes of migrating mammalian cells [19]. Our results are the first to clearly show that CED-10/Rac1 is required for the recycling process, and is not simply a recycling cargo. Furthermore our work provides mechanistic insight into this requirement, showing that CED-10 acts to recruit TBC-2 to endosomal membranes.

These results indicate that down-regulation of RAB-5 by TBC-2 is an important aspect of cargo recycling, and may serve as part of a program for Rab conversion along the recycling pathway. This is consistent with early work on Rab5 indicating that overexpressed Rab5(Q78L) inhibits transferrin recycling in HeLa cells [37]. It remains unclear if direct down-regulation of RAB-7 is also important to promote recycling, since the TBC-2 homolog Armus was originally described as a Rab7 GAP, and *C. elegans* TBC-2 displays some GAP activity toward RAB-7 *in vitro*, albeit at a lower level than its activity toward RAB-5 [26], [27].

In one important respect the *ced-10* mutant phenotype differs from that of *tbc-2* null mutants, in that RAB-7-positive late endosomes appear insensitive to CED-10 activity (Figure S5G and S5H). This suggests that while TBC-2 is generally important for regulating RAB-5, its interaction with CED-10 is mainly important for regulating RAB-5 along the recycling arm of the endocytic pathway. The specificity of the *ced-10* mutant phenotype suggests that TBC-2 maintains some activity and membrane localization that is CED-10 independent, perhaps by direct lipid binding and/or interactions with additional endosomal proteins [28].

The mechanisms that give rise to recycling endosomes are not clear, but most work suggests that recycling endosomes are formed from fission products that leave the early endosome as it matures toward a late endosome [7], [38]. Fission products released from the Trans-Golgi also contribute to the recycling endosome [39]. Once endosomal and Golgi-derived fission products fuse with one another and with pre-existing recycling endosomes, they would be expected to take on new recycling endosome-specific characteristics, including changing their phosphoinositide content and Rab-GTPase activities. Given the plethora of evidence that RAB-10 regulates basolateral recycling in many polarized cells, as first shown in the *C. elegans* intestine, one possibility is that the CED-10/TBC-2 interaction acts to promote a RAB-5 to RAB-10 transition [2], [40], [41]. In addition, endosomes are thought to contain functionally distinct subdomains [42], [43]. The recycling endosome may maintain a RAB-5 positive fusogenic domain for incoming vesicles, and likely maintains multiple distinct tubular budding domains that accumulate outgoing cargo destined for different cellular compartments such as the basolateral plasma membrane, apical plasma membrane, and Golgi [42]. Thus another possible role for CED-10 to TBC-2 signaling is to provide negative feedback from RAB-10 to RAB-5 that maintains distinct RAB-10 and RAB-5 subdomains on the common recycling endosome. Both the Rab transition or subdomain maintenance models are consistent with the partial overlap in localization of RAB-5 and RAB-10 observed on basolateral endosomes of the *C. elegans* intestine, and both models predict that RAB-10 will be involved in recruiting or activating CED-10 and TBC-2 on recycling endosomes [2]. Future work will be directed at testing these models.

Interestingly, previous work in MDCK cells expressing constitutively active Rac1(V12) suggested that Rac1 could play a role in regulating the function of the common recycling endosome [44]. In that work Jou et al. identified a Rac1(V12)-induced morphological defect in recycling endosomes that contain both apical and basolateral cargos. However pulse-chase analysis in Rac1(V12) MDCK cells defined defects in apically-directed common endosome-dependent functions (basolateral-to-apical transcytosis, apical recycling, and apical secretion), but surprisingly found no defect in basolateral recycling. The physiological relevance of these results were unclear because expression of dominant negative Rac1(N17) did not affect recycling, and efficient knockdown methods were not available at that time to directly assay Rac1 loss-of-function. It would be of interest to revisit the MDCK system to investigate more fully the role of Rac1 and Armus in Rab5 down-regulation at the common recycling endosome, directly assaying for phylogenetic conservation of the mechanisms that we have defined here in the *C. elegans* intestine.

Our data connecting CED-10 to TBC-2 also has important implications for phagocytosis/engulfment, since CED-10/Rac1 and TBC-2 are both known to function early in the apoptotic corpse phagocytosis pathway. The potential importance of an interaction between CED-10 and TBC-2 during that process remains unexplored, but a function in promoting endocytic recycling may contribute to lamellipodia formation [28], [45]. RAB-10 has also recently been implicated in regulating phagosome maturation, and thus may be relevant to understanding the phagocytic functions of CED-10/Rac1 and TBC-2 [46].

## Materials and Methods

### General methods and strains

All *C. elegans* strains were derived originally from the wild-type Bristol strain N2. Worm cultures, genetic crosses, and other *C. elegans* husbandry were performed according to standard protocols [47]. Strains were maintained at 20°C. A complete list of strains used in this study can be found in Table S1—under a sub-heading “Transgenic and mutant strains used in this study”.

### Plasmid and transgenic worm strain construction

The CED-10 expression plasmid was created by PCR amplification and Gateway cloning of the *ced-10* cDNA, lacking a start codon, into the Gateway entry vector pDONR221 (Invitrogen, Carlsbad, CA). To create N-terminally tagged GFP or RFP/mCherry transgenes for CED-10 or RAB-5(Q78L) for expression specifically in the worm intestine, Gateway destination vectors were used that contain the promoter region of the intestine-specific gene *vha-6* cloned into the *C. elegans* pPD117.01 vector, a Gateway cassette followed by a GFP or RFP/mCherry coding sequence and then the *unc-119* gene of *C. briggsae*.

To construct C-terminally tagged GFP or mCherry transgenes for CED-5/-12 for expression in the worm intestine, cDNA sequences of *C. elegans ced-5* and *ced-12* lacking a stop codon were cloned individually into Gateway entry vector pDONR221 by PCR and BP reaction, and then transferred into intestinal expression vectors by Gateway recombination cloning LR reaction according to the manufacturer's instructions (Invitrogen, Carlsbad, CA). All plasmids used in this study were sequenced and complete plasmid sequences are available on request.

Low copy integrated transgenic lines for all of these plasmids were obtained using the microparticle bombardment method [48].

### Microscopy and image analysis

Live worms were mounted on 2% agarose pads containing 100 mM tetramisole (MP Biomedicals, OH) in M9 buffer. Most GFP versus mCherry/RFP colocalization experiments were performed on L3 and L4 larvae expressing GFP and mCherry/RFP markers. Young adult hermaphrodites expressing GFP were used for taking confocal images. Images taken in the DAPI channel were used to identify broad-spectrum intestinal autofluorescence caused by lipofuscin-positive lysosome-like organelles [25], [49].

Multi-wavelength fluorescence images were obtained using an Axiovert 200 M (Carl Zeiss MicroImaging, Oberkochen, Germany) microscope equipped with a digital CCD camera (C4742–12ER, Hamamatsu Photonics, Hamamatsu, Japan). Metamorph software version 6.3r2 (Universal Imaging, West Chester, PA) was utilized for image acquisition and Z-stacks of images were deconvolved with AutoDeblur Gold software ver 9.3 (AutoQuant Imaging, Watervliet, NY).

To obtain images of GFP fluorescence without interference from autofluorescence, the spectral fingerprinting function of a Zeiss LSM510 Meta confocal microscope system (Carl Zeiss MicroImaging) was used as described previously [2]. Quantification of confocal images was performed with MetaMorph software version 6.3r2. The same threshold values were used for all images within a given experiment. For each marker comparison, at least six animals were analyzed. Three randomly selected regions of per animal were analyzed using circular regions of defined area. Quantification of fluorescence intensities or object count was performed. The average total intensity or average puncta number was calculated. Student's *t*-test was used to determine the difference between the different groups.

### Preparation of worm extracts, GST protein purification, and pull-downs

Worm extracts were prepared from a mixed-stage population of wild-type and *tbc-2(tm2241); vhIs12[P_vha-6_::GFP::tbc-2+Cb-unc-119]* animals. Worms were grown on egg plates, harvested with 0.1 M NaCl, floated in 30% fresh sucrose solution, and washed three times with 0.1 M NaCl. The worm pellet was frozen in liquid nitrogen and stored at −80°C. Frozen worm pellets were thawed on ice. An equal volume of fresh ice-cold lysis buffer (50 mM HEPES pH 7.4, 1 mM EGTA, 150 mM KCl, 1 mM MgCl_2_, 10% glycerol, 0.2% Triton X-100, Protease inhibitors: Pepstatin, Leupectin, Aprotinin, Sodium Orthovanadate, and PMSF) was added. One mL of the suspension was subjected to 35 strokes in a 2-mL pyrex tissue homogenizer (Thermo Fisher Scientific, Waltham, MA) at 4°C. The suspension was centrifuged twice at 12,000 g for 10 minutes and once for 20 minutes at 4°C. The resulting supernatant was recovered.

The plasmid pGEX-5X-CED-10 was constructed by PCR amplification of *ced-10* from a cDNA template (kindly provided by Dr. Erik Lundquist, University of Kansas) using the primers 5′-GAT CGG ATC CCC CAA GCG ATC AAA TGT GTC GT-3′ and 5′-GAT CCT CGA GTT ACT TGC TCT TTT TGG CTC TTT-3′. The resulting PCR product was cloned in plasmid pGEX-5X-2 (GE Healthcare Life Sciences, Buckinghamshire, England) using the *BamHI* and *XhoI* restriction sites.

To purify the GST proteins, overnight cultures (10 mL) of *BL21 E. coli* transformed with pGEX-5X-2 vectors were diluted 10-fold in 2X YT with 100 mg/mL ampicillin, grown for one hour at 37°C with shaking. Protein expression was induced with 0.5 mM IPTG and shaking at 25°C for 2 hours. The culture was centrifuged at 5,000 g for 10 minutes at 4°C and the pellet was resuspended in 10 mL ice-cold PBS, centrifuged at 3,000 g for 10 minutes at 4°C, and the pellet was resuspended in 1 mL PBS containing 1 mM PMSF and 7 mM ß-mercaptoethanol. Cells were sonicated 10 seconds on ice, freeze/thawed three times in liquid nitrogen and a 20°C water bath, and mixed with 100 µL of 10% Triton X-100. The sample was centrifuged at 12,000 g for 5 minutes at 4°C. The supernatant was gently mixed with 100 µL of 50% glutathione sepharose beads coated with 5% BSA. The sample was centrifuged 500 g for 60 seconds at room temperature and washed with 1 mL of ice-cold PBS two times, centrifuged for 10 seconds at room temperature and the pellet was resuspended in 1 mL PBS was stored at 4°C for not more than 4 weeks.

For the pull-down assays, 10 µg of the purified GST-CED-10 (or GST as the negative control) was added to 800 µL of worm lysate, 20 µL of extra glutathione sepharose beads (3X washed with PBS and pre-coated with 5% BSA), 0.2 mM GDP or GTPγS (Sigma-Aldrich, St. Louis, MO) and 10 mM MgCl_2_, and then incubated for 2 hours rotating at 4°C. The mixture was washed 3 times with ice-cold lysis buffer (without protease inhibitors) and proteins were eluted in 60 µL 2X Laemmli buffer for 20 minutes at 65°C. The samples were centrifuged at 500 g for one minute at room temperature and 25 µL of eluted proteins were carefully collected from the solution above the beads and analyzed by SDS-polyacrylamide-gel electrophoresis using 8% resolving gel and blotted onto nitrocellulose membrane (Bio-Rad Laboratories, Hercules, CA). GFP::TBC-2 was detected using a goat anti-GFP antibody (Rockland Inc., Gilbertville, PA) and a donkey anti-goat antibody (Santa Cruz Biotechnology, CA) at a 1∶1000 and a 1∶10,000 dilution, respectively. To detect GST and GST::CED-10 proteins, the nitrocellulose membrane was reprobed with a rabbit anti-GST antibody and a goat anti-rabbit antibody (Sigma-Aldrich, St. Louis, MO) at a 1∶2000 and a 1∶10,000 dilution, respectively.

## Supporting Information

Figure S1 No change in retrograde recycling cargo protein MIG-14-GFP was observed in *ced-10* and *ced-12* mutants. Representative confocal micrographs of MIG-14(Wntless)-GFP expressed in the intestine of living intact animals, in the indicated genetic backgrounds, are shown (A–F). Average total MIG-14-GFP intensities are shown in (G). Error bars represent standard deviations from the mean (n = 18 each, 6 animals of each genotype sampled in three different regions of each intestine). Scale bar, 10 µm.
(TIF)

Figure S2 Recycling cargo hTfR and hTAC, and recycling endosome markers RME-1 and SDPN-1 accumulate in *ced-5* mutants. (A,B) Recycling cargo hTAC-GFP over-accumulates in *ced-5(n1812)* mutants. (C,D) Recycling cargo hTfR-GFP accumulates in *ced-5(n1812)* mutants. (I) Quantification of hTAC-GFP and hTfR-GFP intensities in the intestine of living wild-type and *ced-5* mutant animals. Asterisks indicate a significant difference in the one-tailed Student's *t* test (***p<0.0001). (E,F) Recycling endosome marker GFP-RME-1 accumulates abnormally in *ced-5(n1812)* mutants. (G,H) Recycling endosome marker SDPN-1-GFP accumulates abnormally in *ced-5(n1812)* mutants. (J) Quantification of GFP-RME-1 and SDPN-1-GFP intensity in the intestine of living wild-type and *ced-5* mutants. Error bars represent standard deviations from the mean (n = 18 each, 6 animals of each genotype sampled in three different regions of each intestine). Scale bar, 10 µm.
(TIF)

Figure S3 No change in the localization or intensity of recycling cargo hTfR and hTAC, or recycling endosome markers RME-1 and SDPN-1, in *ced-2* mutants. (A–H) Recycling cargo hTAC-GFP and hTfR-GFP, and recycling endosome markers GFP-RME-1 and SDPN-1- GFP did not change distribution or intensity in *ced-2(e1752)* mutants. (I–J) Quantification of indicated marker intensities in the intestine of living wild-type and *ced-2* mutant animals. Error bars represent standard deviations from the mean (n = 18 each, 6 animals of each genotype sampled in three different regions of each intestine). Scale bar, 10 µm.
(TIF)

Figure S4 Quantification of endosome marker puncta number in *ced-10* and *ced-12* mutants. Bar graph representation of puncta number, rather than puncta intensity, for the data shown in main Figure 2. Asterisks indicate a significant difference in the one-tailed Student's *t* test (***p<0.0001, **p = 0.001). Error bars represent standard deviations from the mean (n = 18 each, 6 animals of each genotype sampled in three different regions of each intestine).
(TIF)

Figure S5 Further analysis of endosome markers in *ced-10(n3246)* and *ced-12(tp2)* mutants. (A–C) Basolateral recycling endosome marker SDPN-1-GFP over-accumulates in *ced-10(n3246)* and *ced-12(tp2)* mutants. (D–F) Apical recycling endosome marker GFP-RAB-11 was not affected by *ced-10* and *ced-12* mutants. (G–I) Late endosome marker GFP-RAB-7 was not affected by *ced-10(n3246)* and *ced-12(tp2)* mutants. (J) Quantification of SDPN-1-GFP intensity in the intestine of living wild-type, *ced-10*, and *ced-12* mutants. The asterisk indicates a significant difference in the one-tailed Student's T-test (***p<0.0001). (K) Quantification of GFP-RAB-11 and GFP-RAB-7 intensity in the intestine of living wild-type, *ced-10*, and *ced-12* mutants. Error bars represent standard deviations from the mean (n = 18 each, 6 animals of each genotype sampled in three different regions of each intestine). Scale bar, 10 µm.
(TIF)

Figure S6 Further analysis of CED-10 and CED-12 localization in the intestine. (A–C) RFP-CED-10 fails to colocalize with late endosome marker GFP-RAB-7. (D–F) RFP-CED-10 does not co-localize with the Golgi marker AMAN-2/Mannosidase-GFP, but often labels structures juxtaposed to the Golgi ministacks. (G–I) RFP-CED-10 and GFP-HGRS-1 label different endosome types. Virtually no overlap was observed between RFP-CED-10 and GFP-HGRS-1 labeled multivesicular endosomes. (J–L) CED-12-GFP colocalizes with RFP-RME-1 on basolateral recycling endosomes. In each image autofluorescent lysosome-like organelles can be seen in all three channels with the strongest signal in blue, whereas GFP appears only in the green channel and RFP/mCherry only in the red channel. Signals observed in the green or red channels that do not overlap with signals in the blue channel are considered bone fide GFP or RFP/mCherry signals, respectively. Scale bar, 10 µm.
(TIF)

Table S1 Strain list: Summary of the transgenic and mutant strains used during this work.
(DOCX)
