# Supplementary figures and images for: CED-10/Rac1 Regulates Endocytic Recycling through the RAB-5 GAP TBC-2
Source: PLoS Genet. 2012 Jul 12;8(7):e1002785. doi: 10.1371/journal.pgen.1002785 (PMC3395619; doi:10.1371/journal.pgen.1002785)

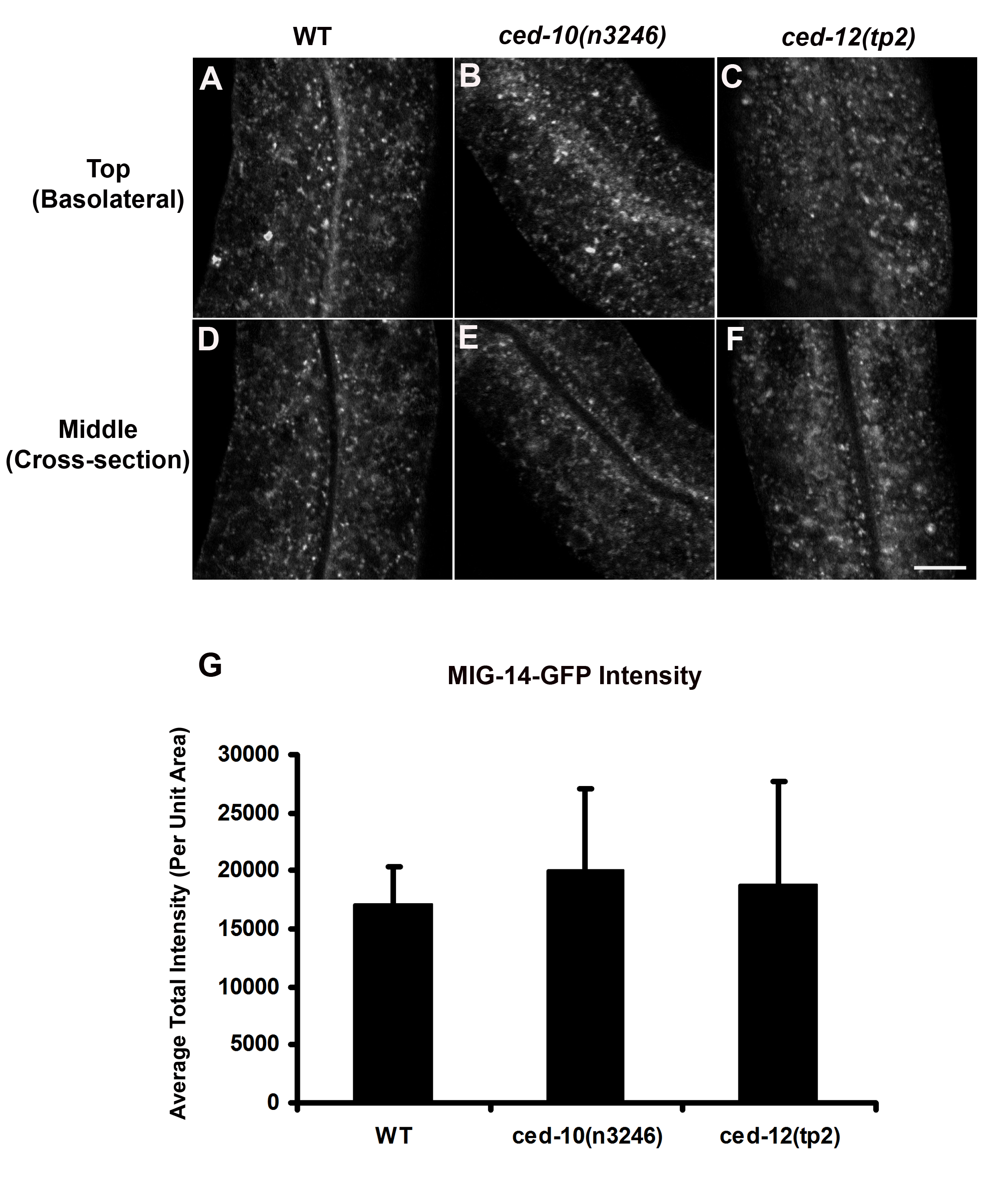

Supplement: Figure S1 — No change in retrograde recycling cargo protein MIG-14-GFP was observed in ced-10 and ced-12 mutants. Representative confocal micrographs of MIG-14(Wntless)-GFP expressed in the intestine of living intact animals, in the indicated genetic backgrounds, are shown (A–F). Average total MIG-14-GFP intensities are shown in (G). Error bars represent standard deviations from the mean (n = 18 each, 6 animals of each genotype sampled in three different regions of each intestine). Scale bar, 10 µm. (TIF) [file pgen.1002785.s001.tif]

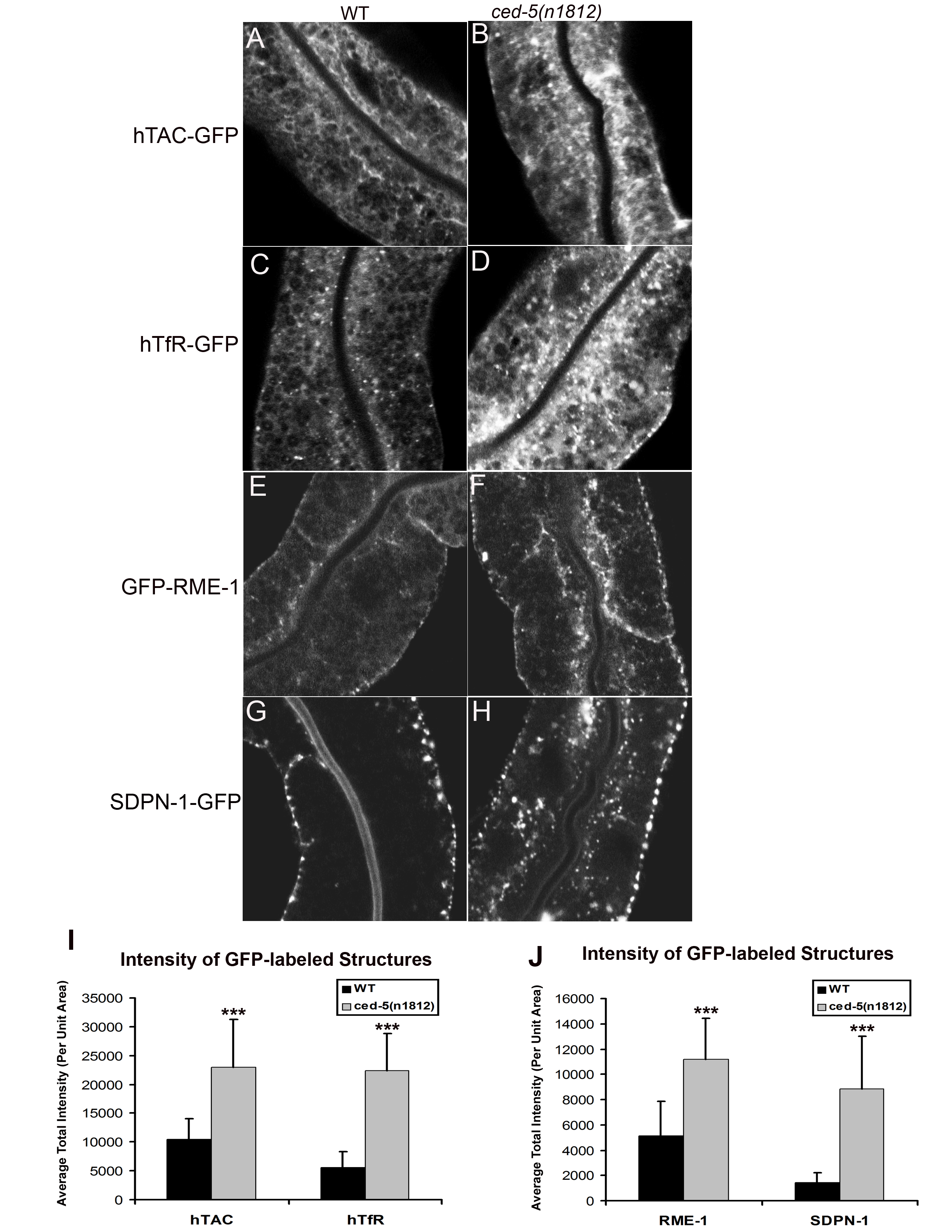

Supplement: Figure S2 — Recycling cargo hTfR and hTAC, and recycling endosome markers RME-1 and SDPN-1 accumulate in ced-5 mutants. (A,B) Recycling cargo hTAC-GFP over-accumulates in ced-5(n1812) mutants. (C,D) Recycling cargo hTfR-GFP accumulates in ced-5(n1812) mutants. (I) Quantification of hTAC-GFP and hTfR-GFP intensities in the intestine of living wild-type and ced-5 mutant animals. Asterisks indicate a significant difference in the one-tailed Student's t test (***p<0.0001). (E,F) Recycling endosome marker GFP-RME-1 accumulates abnormally in ced-5(n1812) mutants. (G,H) Recycling endosome marker SDPN-1-GFP accumulates abnormally in ced-5(n1812) mutants. (J) Quantification of GFP-RME-1 and SDPN-1-GFP intensity in the intestine of living wild-type and ced-5 mutants. Error bars represent standard deviations from the mean (n = 18 each, 6 animals of each genotype sampled in three different regions of each intestine). Scale bar, 10 µm. (TIF) [file pgen.1002785.s002.tif]

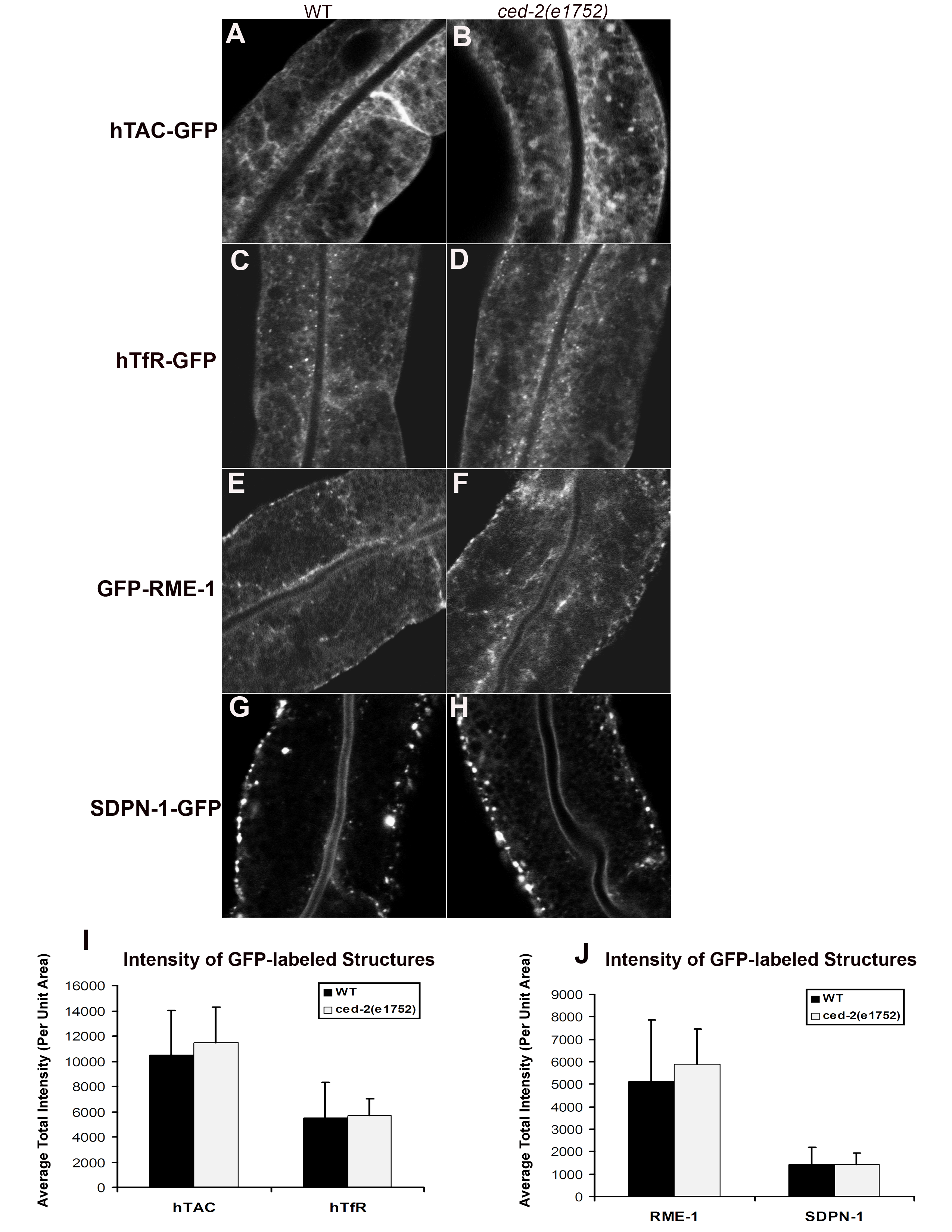

Supplement: Figure S3 — No change in the localization or intensity of recycling cargo hTfR and hTAC, or recycling endosome markers RME-1 and SDPN-1, in ced-2 mutants. (A–H) Recycling cargo hTAC-GFP and hTfR-GFP, and recycling endosome markers GFP-RME-1 and SDPN-1- GFP did not change distribution or intensity in ced-2(e1752) mutants. (I–J) Quantification of indicated marker intensities in the intestine of living wild-type and ced-2 mutant animals. Error bars represent standard deviations from the mean (n = 18 each, 6 animals of each genotype sampled in three different regions of each intestine). Scale bar, 10 µm. (TIF) [file pgen.1002785.s003.tif]

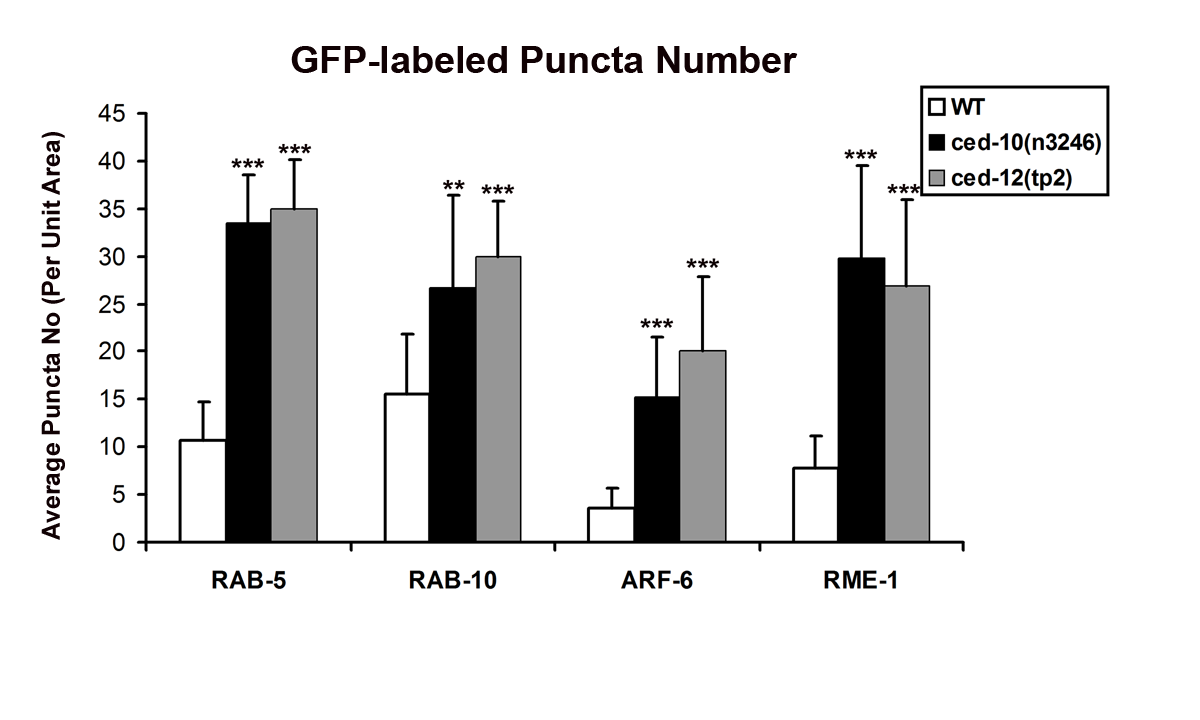

Supplement: Figure S4 — Quantification of endosome marker puncta number in ced-10 and ced-12 mutants. Bar graph representation of puncta number, rather than puncta intensity, for the data shown in main Figure 2. Asterisks indicate a significant difference in the one-tailed Student's t test (***p<0.0001, **p = 0.001). Error bars represent standard deviations from the mean (n = 18 each, 6 animals of each genotype sampled in three different regions of each intestine). (TIF) [file pgen.1002785.s004.tif]

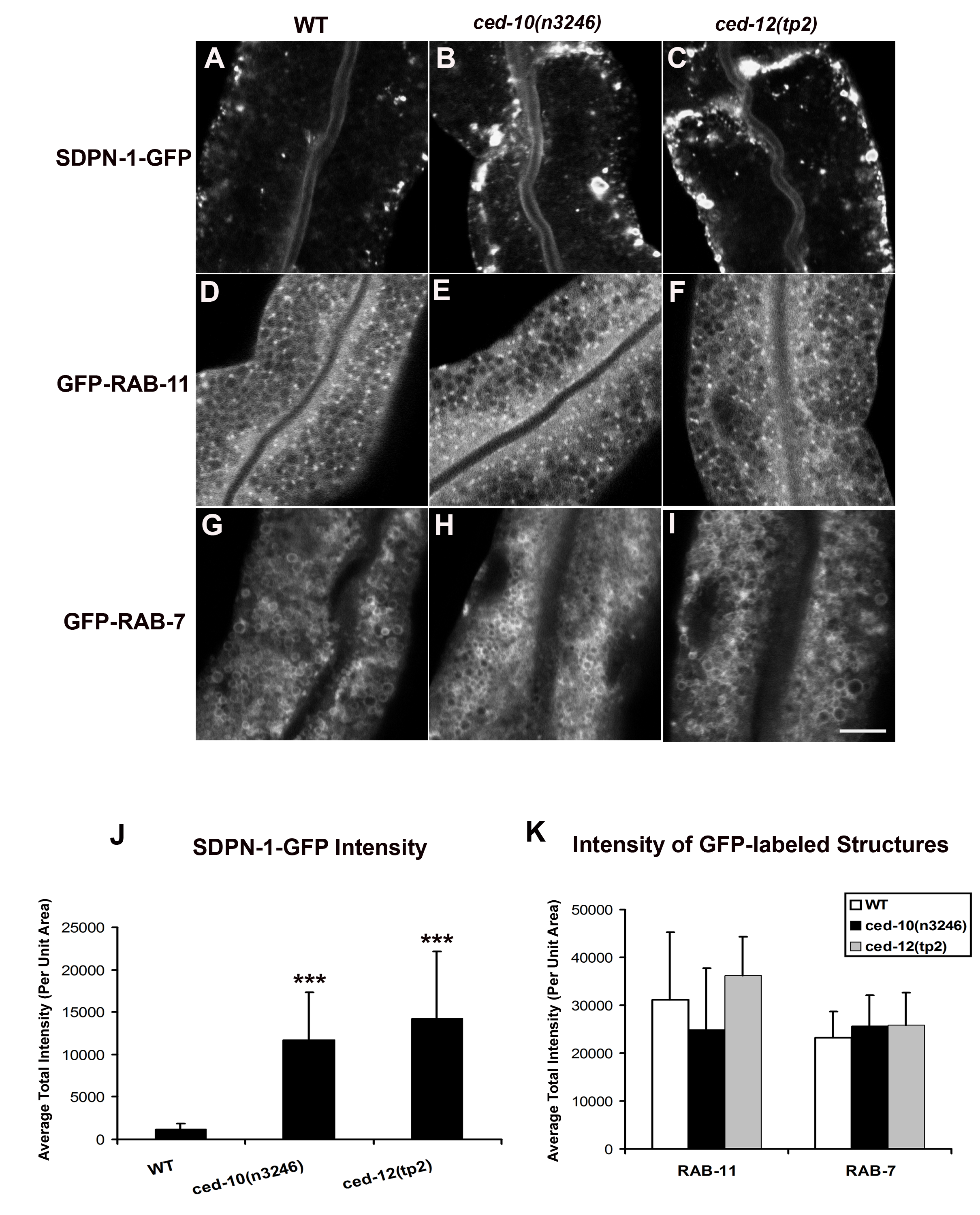

Supplement: Figure S5 — Further analysis of endosome markers in ced-10(n3246) and ced-12(tp2) mutants. (A–C) Basolateral recycling endosome marker SDPN-1-GFP over-accumulates in ced-10(n3246) and ced-12(tp2) mutants. (D–F) Apical recycling endosome marker GFP-RAB-11 was not affected by ced-10 and ced-12 mutants. (G–I) Late endosome marker GFP-RAB-7 was not affected by ced-10(n3246) and ced-12(tp2) mutants. (J) Quantification of SDPN-1-GFP intensity in the intestine of living wild-type, ced-10, and ced-12 mutants. The asterisk indicates a significant difference in the one-tailed Student's T-test (***p<0.0001). (K) Quantification of GFP-RAB-11 and GFP-RAB-7 intensity in the intestine of living wild-type, ced-10, and ced-12 mutants. Error bars represent standard deviations from the mean (n = 18 each, 6 animals of each genotype sampled in three different regions of each intestine). Scale bar, 10 µm. (TIF) [file pgen.1002785.s005.tif]

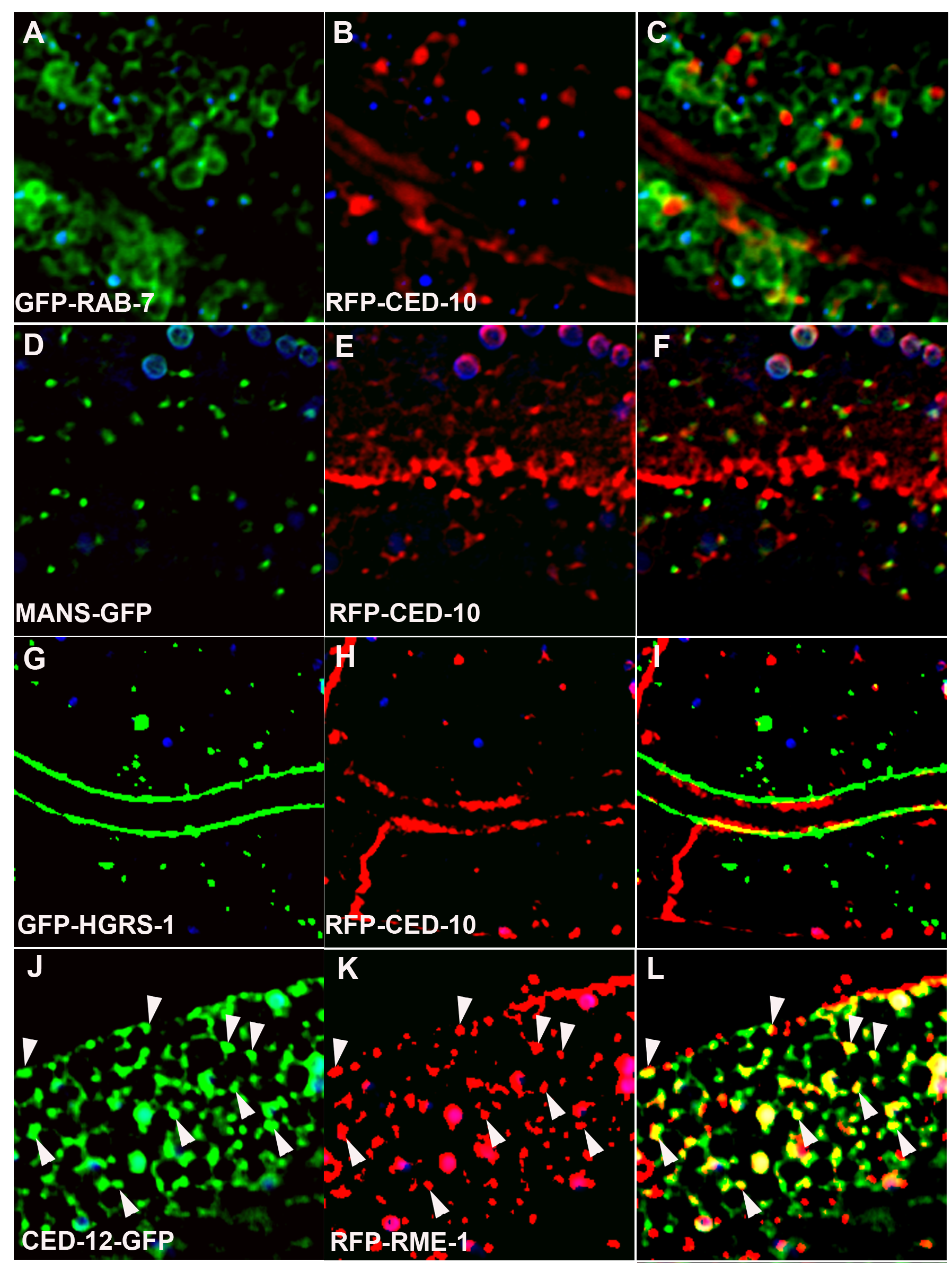

Supplement: Figure S6 — Further analysis of CED-10 and CED-12 localization in the intestine. (A–C) RFP-CED-10 fails to colocalize with late endosome marker GFP-RAB-7. (D–F) RFP-CED-10 does not co-localize with the Golgi marker AMAN-2/Mannosidase-GFP, but often labels structures juxtaposed to the Golgi ministacks. (G–I) RFP-CED-10 and GFP-HGRS-1 label different endosome types. Virtually no overlap was observed between RFP-CED-10 and GFP-HGRS-1 labeled multivesicular endosomes. (J–L) CED-12-GFP colocalizes with RFP-RME-1 on basolateral recycling endosomes. In each image autofluorescent lysosome-like organelles can be seen in all three channels with the strongest signal in blue, whereas GFP appears only in the green channel and RFP/mCherry only in the red channel. Signals observed in the green or red channels that do not overlap with signals in the blue channel are considered bone fide GFP or RFP/mCherry signals, respectively. Scale bar, 10 µm. (TIF) [file pgen.1002785.s006.tif]
