# Supplementary material for: CED-10/Rac1 Regulates Endocytic Recycling through the RAB-5 GAP TBC-2
Source: PLoS Genet. 2012 Jul 12;8(7):e1002785. doi: 10.1371/journal.pgen.1002785 (PMC3395619; doi:10.1371/journal.pgen.1002785)
Supplement: Table S1 — Strain list: Summary of the transgenic and mutant strains used during this work. (DOCX) [file pgen.1002785.s007.docx]

**Transgenic and mutant strains used in this study**

*pwIs112[Pvha-6::hTAC::GFP]* [2]

*pwIs717[Pvha-6::hTfR::GFP]* [2]

*pwIs883[Pvha-6::EHBP-1::mCherry]* [6]

*pwIs859[Pvha-6::mCherry::CED-10]* (this work)

*pwIs807[Pvha-6::CED-12::mCherry]* (this work)

*pwIs72[Pvha-6::GFP::RAB-5]* [2]

*pwIs206[Pvha-6::GFP::RAB-10]* [2]

*pwIs601[Pvha-6::ARF-6::GFP]* (this work)

*pwIs87[Pvha-6::GFP::RME-1]* [2]

*pwIs731[Pvha-6::GFP::CED-10]* (this work)

*pwIs846[Pvha-6::RFP::RAB-5]* [3]

*pwIs852[Pvha-6::RFP::RME-1]* [3]

*pwIs414[Pvha-6::mCherry::RAB-*10*]* (this work)

*pwIs69[Pvha-6::GFP::RAB-11]* [2]

*vhIs12[Pvha-6::GFP::TBC-2]* [27]

*vhIs1[Pvha-6::mCherry::TBC-2]* [27]

*pwIs722[Pvha-6::SDPN-1::GFP]* [3]

*pwIs765[Pvha-6::MIG-14::GFP]* [5]

*pwIs170[Pvha-6::GFP::RAB-7]* [2]

*pwIs481[Pvha-6::MANS::GFP]* [2]

*pwIs518[Pvha-6::GFP::HGRS-1]* [5]

*pwIs770[Pvha-6::CED-12::GFP]* (this work)

*pwIs950[Pvha-6::RFP::RAB-5(Q78L)]* (this work)

*pwIs954[Pvha-6::RFP::RAB-5(Q78L)]* (this work)

*ced-10(n3246)* [15]

*ced-12(tp2)* [14]

*rab-10(q373)* [2]

*rme-1(b1045)* [50]

*ced-5(n1812)* [16]

*ced-2(e1752)* [15]

*tbc-2(tm2241)* [27]

**References**

50. Grant B, Zhang Y, Paupard MC, Lin SX, Hall DH, et al. (2001) Evidence that RME-1, a conserved C. elegans EH-domain protein, functions in endocytic recycling. Nat Cell Biol 3: 573-579.
